# Cerebrospinal Fluid Flow Cytometry in Pediatric Acute Lymphoblastic Leukemia: A Multicenter Retrospective Study in China

**DOI:** 10.1002/cam4.70452

**Published:** 2024-12-09

**Authors:** Songji Tu, Kunlong Zhang, Chenzhu Liu, Ningling Wang, Jinhua Chu, Linhai Yang, Zhiwei Xie, Lingling Hang, Jun Li, Wenyu Yang, Xiaofan Zhu

**Affiliations:** ^1^ Department of Pediatrics The Second Affiliated Hospital of Anhui Medical University Hefei China; ^2^ State Key Laboratory of Experimental Hematology, National Clinical Research Center for Blood Diseases, Institute of Hematology and Blood Diseases Hospital Chinese Academy of Medical Sciences and Peking Union Medical College Tianjin China

**Keywords:** acute lymphoblastic leukemia, cerebrospinal fluid, cumulative incidence of relapse, flow cytometry, multi‐center study, pediatric

## Abstract

**Objective:**

The central nervous system (CNS) is a frequent site of relapse in childhood acute lymphoblastic leukemia (ALL). This study aims to investigate the utility of cerebrospinal fluid (CSF) flow cytometry in detecting CNS infiltration and relapse.

**Methods:**

Flow cytometry was used to detect CSF leukemia cells, and patients were categorized into the CSF Flow+ and CSF Flow− groups. The primary outcome was the cumulative incidence of relapse (CIR) in the CSF Flow+ and CSF Flow− groups.

**Results:**

A total of 1301 patients were enrolled, 159 patients (12.2%) showed positive CSF flow cytometry results. The CNS Flow+ patients exhibited significantly higher rates of any CNS relapse (22.5% vs. 0.2%, *p* < 0.01) and isolated CNS relapse (10.7% vs. 0%, *p* < 0.01) compared to CNS Flow− patients. Cox regression analysis revealed that risk factors for isolated CNS relapse included a positive result of D46 minimal residual disease (MRD) and CSF Flow+ at a non‐initial diagnosis. For any CNS relapse, the significant risk factors were CSF Flow+ at the initial diagnosis and CSF Flow+ at a non‐initial diagnosis.

**Conclusions:**

CSF flow cytometry may have clinical utility in detecting CNS infiltration among pediatric ALL patients. It could contribute to more effective risk stratification and treatment adjustments and potentially reduce the risk of CNS relapse.

## 1 Introduction

Acute lymphoblastic leukemia (ALL) is the most prevalent childhood malignancy, comprising 29% of childhood cancer diagnoses [1]. Despite advances in treatment, ALL remains a significant health concern due to extramedullary infiltration, particularly within the central nervous system (CNS) [2]. CNS involvement at diagnosis is associated with poor prognosis, increased risk of treatment failure, and worse event‐free survival (EFS) and overall survival (OS) [3, 4, 5]. However, detecting CNS infiltration presents challenges and limitations. While routine lumbar puncture is essential for diagnosis, asymptomatic CNS involvement can be missed. Traditional diagnostic methods lack the sensitivity to accurately detect CNS infiltration, highlighting the need for more effective diagnostic strategies.

Advancements in technology and reagents have propelled flow cytometry to the forefront as a potent tool for immunophenotyping, now playing an essential role in diagnosing various forms of leukemia [6]. When combined with histomorphology and additional data, multi‐parameter flow cytometry can rapidly and precisely diagnose leukemia and accurately classify its subtype, per the World Health Organization (WHO) classification standards [7, 8]. Compared to traditional cytological detection, flow cytometry has higher sensitivity and can more accurately detect CNS involvement. Moreover, flow cytometry can detect abnormal blast populations within cerebrospinal fluid (CSF), offer lineage classification of these blasts, and yield prognostic insights [9, 10]. According to research findings, CNS+ patients detected by flow cytometry were significantly associated with worse outcomes compared to CNS− patients [11, 12]. Therefore, the application of flow cytometry was recommended by many researchers. Arber et al. suggested performing flow cytometry on CSF samples of patients with ALL [11].

Therefore, this study aims to investigate the utility of CSF flow cytometry in detecting CNS infiltration and relapse.

## 2 Methods

### 2.1 Study Design and Participants

This retrospective study was conducted at the Second Affiliated Hospital of Anhui Medical University (Department of Paediatric Haematology and Oncology) and the Institute of Haematology and Blood Diseases Hospital, under the Chinese Academy of Medical Sciences and Peking Union Medical College between January 2015 and October 2020. The inclusion criteria were: (1) aged below 18 years old; (2) newly diagnosed ALL patients enrolled in the CCCG‐ALL‐2015 protocol; (3) CSF was done concurrently with routine, smear, and flow tests during treatment. Patients who received their initial diagnosis and treatment for relapse at an external facility were excluded. This study was approved by the ethics committee of the Second Affiliated Hospital of Anhui Medical University (PJ‐YX201501). All subjects signed the written informed consent.

### 2.2 Data Collection and Definition

Demographic data, including sex, age, and examination data such as white blood cell (WBC) count and immunophenotype, were derived from each center's electronic medical systems.

### 2.3 CSF Analysis and Treatments

Fresh CSF samples were processed without preservatives. Specimens should be sent for testing immediately after collection, ideally within 2 h at the latest. A CSF Flow+ result is defined by the presence of cells with an abnormal phenotype via flow cytometry. FCM analysis was conducted utilizing a FACS Canto II Cytometer from BD Systems and evaluated using Infinicyte Software version 2.0 from Cytognos S.L., based in Salamanca, Spain. A minimum of three leukemic cells exhibiting a leukemia‐associated phenotype was necessary to determine CNS positivity. Cases with traumatic taps were included in the analysis if the sample could provide valid FCM results.

At the initial diagnosis, a positive CSF flow cytometry result necessitates a treatment protocol that starts with five intrathecal injections. During the consolidation‐maintenance treatment phase, a positive CSF flow cytometry finding, accompanied by a negative bone marrow assessment, mandates an escalation of intrathecal injections. This escalated treatment continues until flow cytometry confirms the CSF is clear of abnormal cells, after which two additional intrathecal injections are administered. In the case of concurrent disease recurrence in other areas, a regimen of combined systemic chemotherapy is indicated. Complete remission (CR) was defined as the presence of less than 5% primitive cells in bone marrow smears without evidence of extramedullary infiltration. A relapse was defined as the appearance of primitive cells exceeding 5% in bone marrow smears or the development of extramedullary disease after CR [13].

### 2.4 Outcomes

The primary outcome was the cumulative incidence of relapse (CIR) in the CSF Flow+ and CSF Flow− groups. The secondary outcomes included the CIR within the CSF Flow+ group during various treatment phases.

### 2.5 Statistical Analysis

Data analysis was performed using SPSS 24.0 (IBM Corp., Armonk, NY, USA). Continuous data with a normal distribution were described as mean ± standard deviation (SD) and analyzed using Student's *t*‐test; otherwise, they were presented as medians (interquartile range, IQR) and analyzed using the Wilcoxon rank‐sum test. Categorical variables were presented as *n* (%) and analyzed using the chi‐squared or Fisher's exact test. The 6‐year CIR was analyzed using the Kaplan–Meier method and Gray's test. Prognostic factors were examined by Cox proportional hazard regression models. Two‐sided *p*‐values of < 0.05 were considered statistically significant (two‐tailed testing).

## 3 Results

**FIGURE 1:**
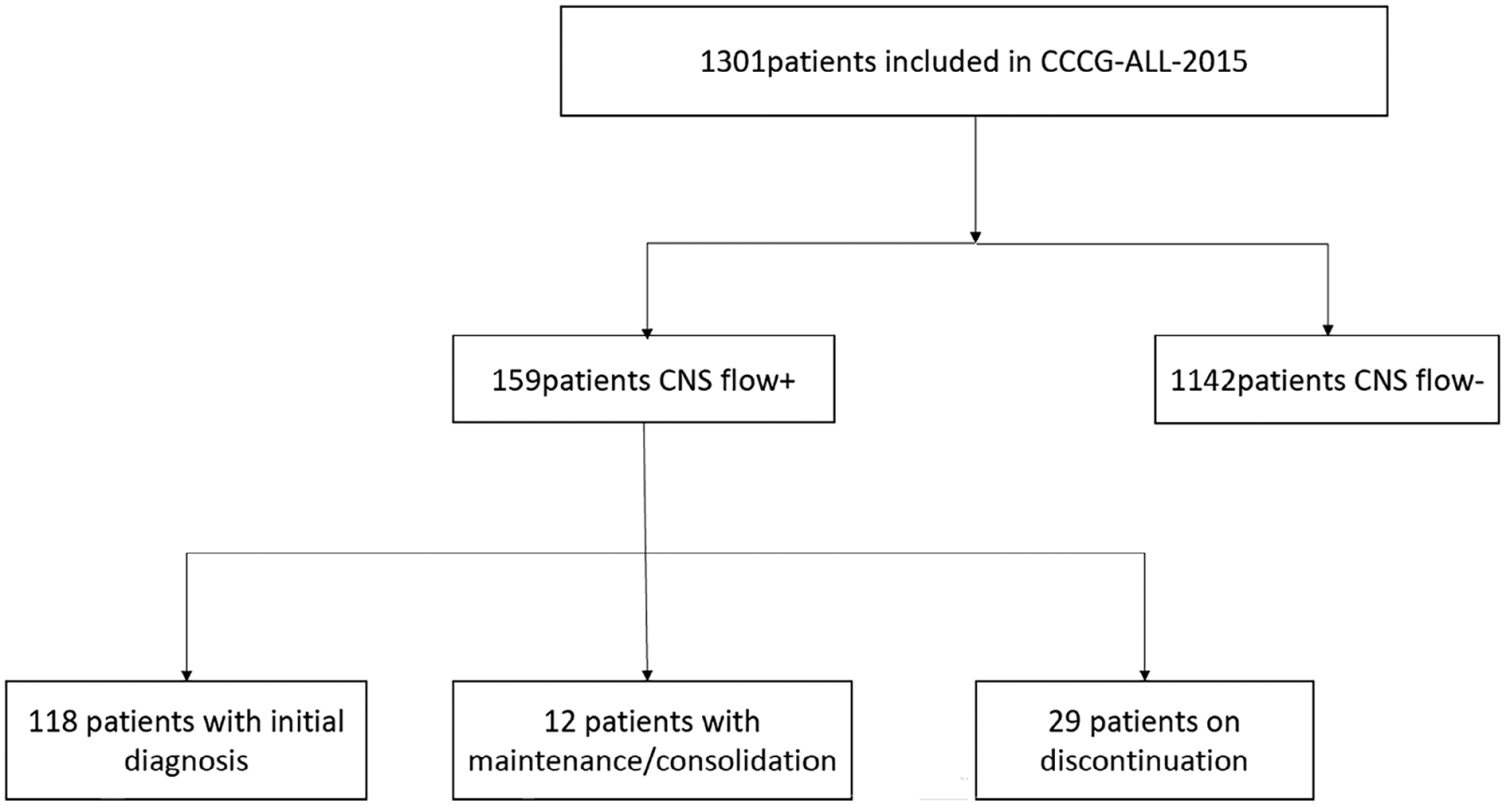
Study flowchart.

A total of 1301 patients were enrolled, 159 patients (12.2%) showed positive CSF flow cytometry results. Of these, 118 (74.2%) were detected during the initial diagnosis, 12 (7.6%) manifested during the consolidation‐maintenance phase, and 29 (18.2%) were detected when medication was discontinued (Figure 1). During the consolidation‐maintenance phase and after discontinuation, all participants who tested positive for flow cytometry had initial negative flow cytometry at the first diagnosis. The age and gender distribution were comparable between CSF Flow+ and CSF Flow− patients (all *p* > 0.05). However, CSF Flow+ patients were more likely to have significantly higher WBC counts (37.5 (0.6–846.5) vs. 9.7 (0.74–781.6), *p* < 0.01), a higher proportion of T‐cell acute lymphoblastic leukemia (T‐ALL) (19.5% vs. 8.7%, *p* < 0.01), a lower proportion of B‐cell acute lymphoblastic leukemia (B‐ALL) (80.5% vs. 91.3%, *p* < 0.01), and a higher ratio of positive D46MRD (*p* = 0.02) (Table 1).

**TABLE 1:** The clinical characteristics of the CSF Flow+ group and the CSF Flow− group.

Variables	Flow+ (*n* = 159)	Flow− (*n* = 1142)	*p*
Sex, male, *n* (%)	100 (62.9)	668 (58.5)	0.291
Age, median (range), years	5.0 (0.1–15.3)	5.1 (0.6–16.7)	0.807
WBC, median (range), 10^9^/L	37.5 (0.6–846.5)	9.7 (0.74–781.6)	< 0.01
Immunophenotype, *n* (%)			< 0.01
B‐ALL	128 (80.5)	1043 (91.3)	
T‐ALL	31 (19.5)	99 (8.7%)	
D46MRD, *n* (%)			0.02
Positive	27 (17.0)	183 (16.0)	
Negative	126 (79.2)	947 (82.9)	
Missing	6 (3.8)	12 (1.1)	

Abbreviations: ALL, acute lymphoblastic leukemia; WBC, white blood cell.

There were no significant differences in age, gender ratio, WBC count, and D46MRD among CSF Flow+ patients at different treatment stages, including the diagnosis stage, consolidation‐maintenance stage, and discontinuation stage (all *p* > 0.05). However, there were significant differences in immunophenotype distribution among treatment stages (*p* = 0.018) (Table 2).

**TABLE 2:** The clinical characteristics of the CSF Flow+ group at different stages of treatment.

Variables	Diagnosis (*n* = 118)	Consolidation/maintenance (*n* = 12)	Discontinuation (*n* = 29)	*p*
Sex, male, *n* (%)	72 (61.0)	6 (50.0)	21 (72.4)	0.347
Age, median (range), years	4.9 (0.1–15.3)	9.2 (0.8–14.0)	4.5 (1.4–14.9)	0.107
WBC, median (range), 10^9^/L	30.9 (0.6–846.5)	28.8 (3.0–425.8)	47.3 (0.8–800.6)	0.580
Immunophenotype, *n* (%)				0.018
B‐ALL	99 (83.9)	6 (50.0)	23 (79.3)	
T‐ALL	19 (16.1)	6 (50.0)	6 (20.7)	
D46MRD, *n* (%)				0.769
Positive	23 (19.5)	1 (8.3)	4 (13.8)	
Negative	91 (77.1)	11 (91.7)	24 (82.8)	
Missing	4 (3.4)	0	1 (3.4)	

Abbreviations: ALL, acute lymphoblastic leukemia; WBC, white blood cell.

**FIGURE 2:**
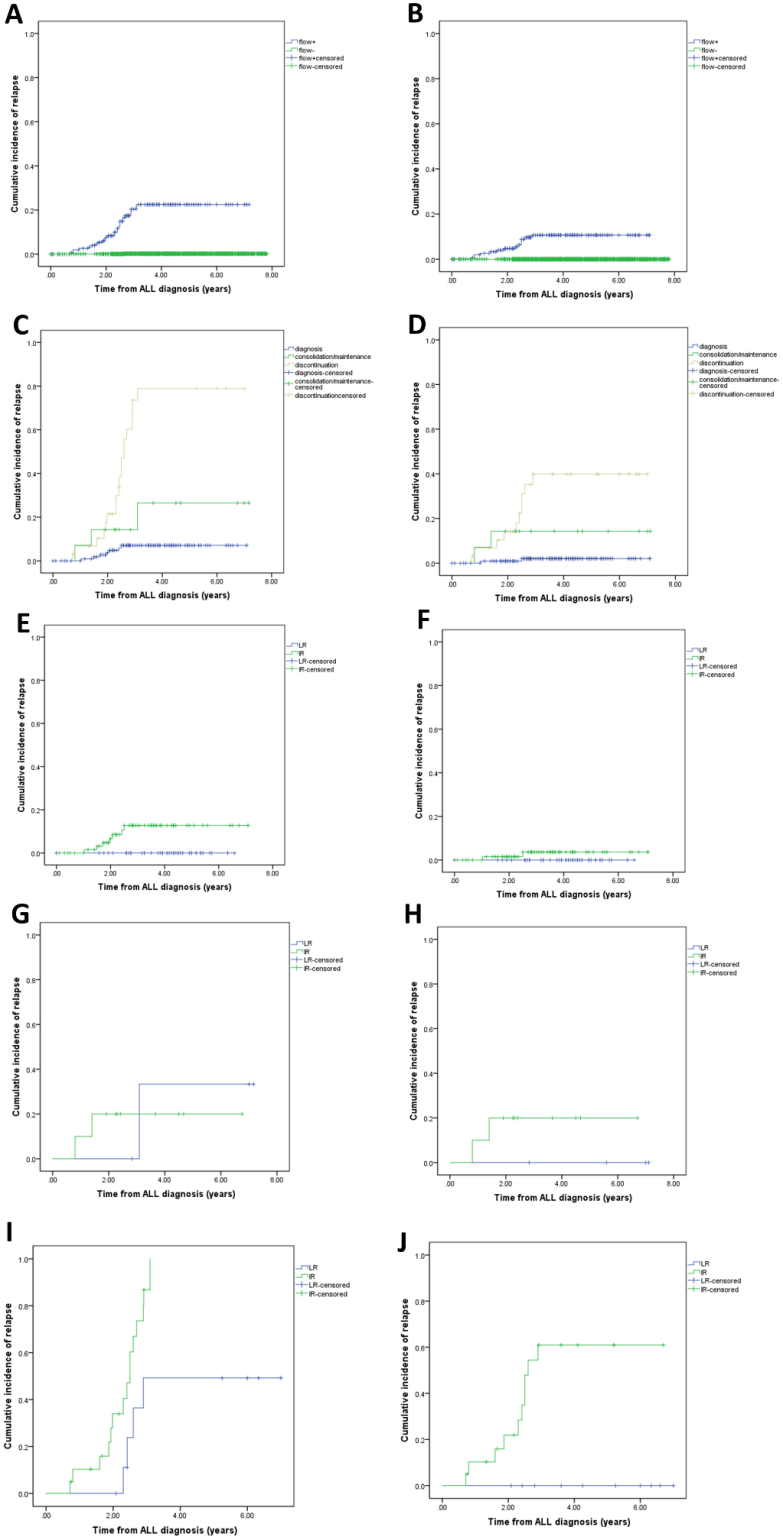
Cumulative Incidence of Relapse (CIR) rates. (A, B) CIR between CSF Flow+ group and Flow− group. (A) CIR involving CNS (22.5% vs. 0.2%, *p* < 0.01). (B) CIR for CNS only (10.7% vs. 0%, *p* < 0.01). (C, D) CIR in the Flow+ group at different stages of treatment. (C) CIR involving CNS (7.1%, 26.5%, 78.9%, *p* < 0.01). (D) CIR for CNS only (1.8%, 14.3%, 39.9%, *p* < 0.01). (E–J) CIR between low‐risk (LR) and intermediate‐risk (IR) groups at different stages of treatment in the Flow+ group. (E) CIR involving CNS in the Flow+ group at initial diagnosis. (F) CIR for CNS only in the Flow+ group at initial diagnosis. (G) CIR involving CNS in the Flow+ group at consolidation‐maintenance. (H) CIR for CNS only in the Flow+ group at consolidation‐maintenance. (I) CIR involving CNS in the Flow+ group after discontinuation. (J) CIR for CNS only in the Flow+ group after discontinuation.

The CNS Flow+ patients exhibited significantly higher rates of any CNS relapse (22.5% vs. 0.2%, *p* < 0.01) and isolated CNS relapse (10.7% vs. 0%, *p* < 0.01) compared to CNS Flow− patients (Figure 2A,B). In the CSF Flow+ group, the 6‐year CIR for isolated CNS relapse increased progressively from 1.8% (95% CI: 1.6–2.0) at diagnosis to 14.3% (95% CI: 12.4–16.2) during maintenance‐consolidation, and further to 39.9% (95% CI: 38.7–41.1) following treatment discontinuation (*p* < 0.01). Similarly, the CIR for any CNS relapse escalated from 7.1% (95% CI: 7.0–7.2) at diagnosis to 26.5% (95% CI: 25.2–27.8) during maintenance‐consolidation and spiked to 78.9% (95% CI: 77.2–80.6) following treatment discontinuation (*p* < 0.01) (Figure 2C,D). In the CSF Flow+ group, for newly diagnosed patients, the CIR for CNS relapse was no difference between low‐risk (LR) and intermediate‐risk (IR) groups for any CNS (0% vs. 7.9%, *p* = 0.093) and isolated CNS (0% vs. 2.3%, *p* = 0.379) (Figure 2E,F).

During consolidation‐maintenance, there was no significant difference in CIR for any CNS between LR and IR (33.3% vs. 25.0%, *p* = 0.881), and for isolated CNS (0% vs. 25.0%, *p* = 0.301) (Figure 2G,H). Post‐treatment discontinuation, CIR also showed significant differences between the LR and IR groups for any CNS (55.0% vs. 91.3%, *p* = 0.015) and for isolated CNS (0% vs. 60.9%, *p* = 0.006) (Figure 2I,J). The recurrence rate for any CNS and isolated CNS were 2.6% (95% CI: 2.5–2.7) and 1.2% (95% CI: 1.1–1.3), respectively. Cox proportional hazard regression models revealed that risk factors for isolated CNS relapse included a positive result of D46MRD (HR: 0.002, 95% CI: 0–0.937, *p* = 0.048) and CSF Flow+ at a non‐initial diagnosis (HR: 29.875, 95% CI: 1.007–886.041, *p* = 0.045). For any CNS relapse, the significant risk factor was CSF Flow+ at initial diagnosis (HR 4.985, 95% CI: 1.608–15.451, *p* = 0.005) and CSF Flow+ at a non‐initial diagnosis (HR: 165.215, 95% CI: 57.295–476,410, *p* < 0.01) (Table 3).

**TABLE 3:** Cox proportional hazard regression models of CNS‐only or CNS‐involving relapse.

Outcome	Variables	Hazard ratio (95% CI)	*p*
CNS‐only relapse	Male	0.897 (0.026–30.997)	0.952
	Age ≥ 10 years	0.928 (0.015–59.010)	0.972
	WBC ≥ 50 × 10^9^/L	0.919 (0.015–56.809)	0.968
	T‐ALL	1.613 (0.017–154.309)	0.837
	D46MRD ≥ 0.01%	0.002 (0–0.937)	0.048
	CSF Flow+ at initial diagnosis	1.062 (0.03–412.960)	0.984
	CSF Flow+ at non‐initial diagnosis	29.875 (1.007–886.041)	0.045
CNS‐involving relapse	Male	0.513 (0.167–1.575)	0.243
	Age≥ 10 years	2.143 (0.769–5.973)	0.145
	WBC≥ 50 × 10^9^/L	0.503 (0.168–1.510)	0.220
	T‐ALL	1.861 (0.637–5.437)	0.256
	D46MRD ≥ 0.01%	0.174 (0.016–1.897)	0.151
	CSF Flow+ at initial diagnosis	4.985 (1.608–15.451)	0.005
	CSF Flow+ at non‐initial diagnosis	165.215 (57.295–476.410)	< 0.01

## 4 Discussion

This study found that patients with CSF Flow+ cytometry had higher WBC counts and were more likely to have T‐cell ALL. The 6‐year CIR for CNS relapse was significantly higher in the CSF Flow+ group, especially following treatment discontinuation. We also demonstrated that risk factors for CNS relapse include D46MRD and CSF Flow+ cytometry, with the latter being significant at initial diagnosis or not for any CNS recurrence and at non‐initial diagnosis for isolated CNS recurrence.

The study revealed a CSF Flow+ rate of 12.2% (159/1301), surpassing traditional detection methods and aligning with previous reports [14, 15, 16]. Given the demonstrated high sensitivity of flow cytometry in detecting CSF, it is well‐established that it can effectively complement traditional detection methods [17]. This study confirmed that patients with CSF Flow+ status exhibited higher WBC counts and T‐cell ALL percentages, corroborating the results of former investigations [18, 19, 20]. Notably, the MRD levels at Day 46 were more significant in the CSF Flow+ group in our study, a finding that diverges from the results reported in some other studies [21].

It is crucial to recognize the interconnectedness of the CNS and bone marrow in the leukemia process, as they are not isolated conditions. A multi‐center study in Italy revealed that CSF Flow+ is a crucial prognostic indicator [22]. Other studies have demonstrated that the recurrence rate was higher in patients with CSF Flow+, with certain findings reaching statistical significance [19]. However, some findings did not achieve statistical significance and were attributed to the small size of the study cohort [23, 24]. Our study demonstrated that the 6‐year CIR for any CNS relapse and isolated CNS relapse significantly differed between the CSF Flow+ and CSF Flow− groups.

Previous research has demonstrated that CSF Flow+ is linked to CNS recurrence at different time intervals [25]. Our study indicated that the presence of CSF Flow+ at varying stages of treatment correlated with notable variances in CIR, both for any CNS and isolated CNS. This implied the significance of monitoring minimal residual disease in the CSF during treatment.

This study examined a CSF flow cytometry‐guided treatment strategy. Analysis revealed that among patients in the CSF Flow+ group at initial diagnosis, the 6‐year CIR for isolated CNS and any CNS relapse were similar across different risk level subgroups. These results indicated that the adoption of flow cytometry‐informed therapy may lead to a significant reduction in CNS recurrence. During the consolidation‐maintenance phase, our study found no significant differences in the CIR between isolated CNS and any CNS relapses across various risk subgroups. This underscores the potential benefits of CSF‐directed therapy. While there appeared to be a higher CIR for any CNS relapse in the LR group compared to the IR group, the relevance of this trend is uncertain due to the small patient sample size. The discontinuation phase showed notable differences in the CIR for isolated CNS relapse and any CNS relapse among risk subgroups. Importantly, the LR group exhibited no isolated CNS relapse. These findings suggested that flow cytometry‐guided interventions can significantly reduce CNS relapse in LR patients.

Children who have recently been diagnosed with CNS leukemia, experienced traumatic lumbar puncture, and have high levels of WBC have been previously documented as being at high risk for a relapse in the CNS [26]. Additionally, several genetic abnormalities, including BCR‐ABL1, MLL rearrangements, subploidy, and TCF3‐PBX1 fusions, are also considered to pose an increased risk for a relapse in the CNS [27, 28]. A recent study conducted by the NOPHO group revealed that CSF flow positivity at the time of diagnosis was an independent risk factor for relapse in children and adolescents with ALL [29].

Our research revealed that while initial CSF Flow+ status was associated with any CNS recurrence, isolated CNS relapse correlated with elevated D46MRD and non‐primary CSF Flow+. This suggests that children initially identified as CSF Flow+ are less likely to experience isolated CNS relapse when treated with flow‐driven therapy.

The patient in this study was the only one in the CCCG‐ALL‐2015 study to have CSF tested with the simultaneous addition of flow cytometry. The recurrence rate for any CNS and isolated CNS was 2.6% (95% CI: 2.5–2.7) and 1.2% (95% CI: 1.1–1.3), respectively, lower than those reported in the CCCG‐ALL‐2015 study [30, 31]. This is likely due to the targeted intervention based on CSF flow cytometry findings in our cohort, highlighting the benefits of personalized treatment strategies.

However, this study had several limitations. First, the sample size is small, which may introduce bias into the analysis results. Second, there was an imbalance in the number of CSF Flow+ and CSF Flow− patients, and a larger cohort of CSF Flow+ patients would be beneficial for further analysis.

In conclusion, this study found that CSF Flow+ status was associated with a higher risk of CNS relapse, demonstrating the clinical significance of CSF flow cytometry in pediatric ALL. However, larger cohort studies are needed to validate these findings and optimize treatment protocols for pediatric ALL patients.

## Author Contributions

**Songji Tu:** data curation (equal), funding acquisition (equal), writing – original draft (equal), writing – review and editing (equal). **Kunlong Zhang:** funding acquisition (equal), validation (equal), writing – original draft (equal). **Chenzhu Liu:** methodology (equal), writing – original draft (equal). **Ningling Wang:** conceptualization (equal), resources (equal). **Jinhua Chu:** data curation (equal), funding acquisition (equal). **Linhai Yang:** data curation (equal). **Zhiwei Xie:** data curation (equal). **Lingling Hang:** data curation (equal). **Jun Li:** data curation (equal). **Wenyu Yang:** conceptualization (equal). **Xiaofan Zhu:** conceptualization (equal).

## Conflicts of Interest

The authors declare no conflicts of interest.

## Data Availability

The data that support the findings of this study are available from the corresponding author upon reasonable request.
